# Editorial: NAFLD treatment in diabetes: from current dietary interventions to novel treatment

**DOI:** 10.3389/fendo.2025.1572770

**Published:** 2025-03-17

**Authors:** Riccardo Nevola, Giuseppina Costabile, Giuseppe Della Pepa

**Affiliations:** ^1^ Liver Unit, Azienda Ospedaliera di Rilevo Nazionale (AORN) San Giuseppe Moscati, A. Landolfi Hospital, Avellino, Italy; ^2^ Department of Clinical Medicine and Surgery, University of Naples Federico II, Naples, Italy; ^3^ Endocrine Disease Center and Regional Diabetes Center, Azienda Socio-Sanitaria Territoriale (ASST), Bergamo, Italy

**Keywords:** MASLD, type 1 diabetes, type 2 diabetes, glucose metabolism, treatment

Metabolic dysfunction-associated steatotic liver disease (MASLD), formerly known as non-alcoholic fatty liver disease, represents the most common liver disease worldwide, affecting approximately 20–30% of the general population. The histopathological and clinical abnormalities of the MASLD spectrum range from the accumulation of triglycerides in the liver to non-alcoholic steatohepatitis, which may progress to liver fibrosis and advanced cirrhosis. The association between MASLD and diabetes is well established, and there appears to exist an intricate interrelationship whereby the existence of one drives progression to the other. Diabetes seems to be the most important risk factor for MASLD and the most important clinical predictor of the advanced forms of MASLD; on the other hand, MASLD is associated with a worse metabolic profile and a higher prevalence of microvascular and macrovascular complications of DM, independently of other known risk factors.

This Research Topic aimed to provide insight into several aspects of the connection between MASLD and diabetes, presenting novelties on the potential mechanisms and associated factors (nutritional and metabolic) at the basis of these relationships and suggesting therapeutic potential approaches for the treatment of MASLD in diabetes. Overall, four original articles and a review have been published in this Research Topic.

Glucose and lipid metabolism in the liver deeply impact MASLD. To this regard, Zhang Z. et al. reviewed the specific role of the glucokinase regulator gene (GCKR) and the glucokinase regulatory protein (GKRP) in the pathophysiology of MASLD. The GCKR, responsible for encoding the GKRP, acts as a regulator and protector of the glucose-metabolizing enzyme glucokinase (GK) in the liver. Emerging evidence suggests that *GCKR* gene polymorphisms contribute to the pathogenesis and progression of MASLD through synergistic effects with metabolic risk factors. Based on various genetic and biological studies, excessive activation of hepatic GK, by deteriorating GK activity, might promote abnormal glucose uptake, stimulating lipogenesis through multiple pathways, leading to the development and progression of MASLD. Conversely, evidence from GKRP overexpression suggests that its effect on balancing GK activation and restriction may be a more comprehensive therapeutic strategy, enhancing postprandial glucose metabolism, ultimately helping to reverse chronic hyperglycaemia, and restoring metabolic flexibility. Collectively, this review sheds new light on the complex interaction between genes and the environment in MASLD, focusing on the GCKR gene. By integrating evidence from genetics, biology, and drug development, authors reassess the therapeutic potential of targeting GK or GKRP for MASLD treatment, accordingly a holistic strategy for restoring glucose and lipid metabolic balance.

It is widely known that MAFLD is strongly associated with obesity and higher BMI; however, a growing body of research suggests that MAFLD can also occur in non-obese people, especially in the Asian population. Zhang Y. et al. investigated the metabolic characteristics and body composition of non-obese individuals with MAFLD compared with obese MAFLD patients and non-obese healthy controls. Authors showed that the prevalence of MAFLD in non-obese individuals was 13.9%, non-obese MAFLD was older than obese patients. Interestingly, although patients with non-obese MAFLD have lower waist circumference and BMI than those with obese MAFLD, they have a higher incidence of diabetes. Furthermore, GGT and HDL-C were significantly associated with non-obese MAFLD subjects when compared with non-obese healthy controls, suggesting that HDL-c and GGT might be predictors of disease progression in MAFLD patients.

The association between type 1 diabetes and MASLD incidence, particularly in advanced type 1 diabetes with complications, and its genetic predisposition remains contentious. Tuo et al. investigated the causal relationships between type 1 diabetes with various complications and MASLD using a two-sample MR framework, with validation in separate databases. Through an in-depth analysis of a substantial and independent cohort, authors suggest that there may not be a direct causal relationship between type 1 diabetes and MASLD; furthermore, regardless of complications, the genetic susceptibility of type 1 diabetes does not increase the likelihood of MASLD. These findings carry noteworthy implications for understanding the relationships between type 1 diabetes and MASLD, potentially facilitating the clinical management of patients with type 1 diabetes and concurrent MASLD.

Xia et al. explored for the first time the relationship between serum iron status and all-cause mortality in individuals with MASLD in a wide cohort from the Third National Health and Nutrition Examination Survey (NHANES III), including people with DM. Authors show that high serum iron and transferrin saturation were significantly associated with reduced all-cause mortality (20-40%) in a linear pattern. These findings suggest that serum iron and transferrin saturation have the potential to serve as independent biomarkers of all-cause mortality in patients with MASLD and imply the therapeutic potential of modifying iron status.

Immunity and inflammation may be key factors in the evolution of MASLD in people with DM. Yang et al. explored the association between the fasting blood glucose/glycated haemoglobin (FBG/HbA1c) ratio and mortality outcomes in individuals with DM or pre-diabetes, with a particular focus on the potential mediating role of immunity and inflammation evaluated by the Systemic Immune Inflammation (SII) index, which takes into account neutrophil, lymphocyte, and platelet counts.

By a comprehensive analysis of NHANES 1999-2018, authors showed for the first time that inflammation mediated the association of FPG/HbA1c ratio with cardiovascular diseases and all-cause mortality in the population with DM or pre-diabetes. A U-shaped association between FPG/HbA1c ratio and mortality was observed, with critical thresholds identified at 1.080 for cardiovascular disease mortality and 1.013 for all-cause mortality. These findings provide novel insights into the relationship between the FPG/HbA1c ratio, mortality, and the mediating role of inflammation, highlighting the need for a nuanced understanding of glucose metabolism in these populations and suggesting potential targets for intervention to improve outcomes.

Taken together, the studies published in this Research Topic provide the reader with an increased understanding of interactions between MASLD and diabetes, opening new possible scenarios in the evaluation and treatment of subjects suffering from the coexisting MASLD and diabetes.

